# Eye-movement analysis on facial expression for identifying children and adults with neurodevelopmental disorders

**DOI:** 10.3389/fdgth.2023.952433

**Published:** 2023-02-16

**Authors:** Kota Iwauchi, Hiroki Tanaka, Kosuke Okazaki, Yasuhiro Matsuda, Mitsuhiro Uratani, Tsubasa Morimoto, Satoshi Nakamura

**Affiliations:** ^1^ Augmented Human Communication Laboratory, Nara Institute of Science and Technology, Ikoma, Nara, Japan; ^2^ Department of Psychiatry, Nara Medical University School of Medicine, Kashihara, Nara, Japan; ^3^ Osaka Psychiatric Medical Center, Osaka, Japan

**Keywords:** autism spectrum disorder, schizophrenia, convolutional neural networks, eye movement, facial emotion recognition

## Abstract

Experienced psychiatrists identify people with autism spectrum disorder (ASD) and schizophrenia (Sz) through interviews based on diagnostic criteria, their responses, and various neuropsychological tests. To improve the clinical diagnosis of neurodevelopmental disorders such as ASD and Sz, the discovery of disorder-specific biomarkers and behavioral indicators with sufficient sensitivity is important. In recent years, studies have been conducted using machine learning to make more accurate predictions. Among various indicators, eye movement, which can be easily obtained, has attracted much attention and various studies have been conducted for ASD and Sz. Eye movement specificity during facial expression recognition has been studied extensively in the past, but modeling taking into account differences in specificity among facial expressions has not been conducted. In this paper, we propose a method to detect ASD or Sz from eye movement during the Facial Emotion Identification Test (FEIT) while considering differences in eye movement due to the facial expressions presented. We also confirm that weighting using the differences improves classification accuracy. Our data set sample consisted of 15 adults with ASD and Sz, 16 controls, and 15 children with ASD and 17 controls. Random forest was used to weight each test and classify the participants as control, ASD, or Sz. The most successful approach used heat maps and convolutional neural networks (CNN) for eye retention. This method classified Sz in adults with 64.5% accuracy, ASD in adults with up to 71.0% accuracy, and ASD in children with 66.7% accuracy. Classifying of ASD result was significantly different (p<.05) by the binomial test with chance rate. The results show a 10% and 16.7% improvement in accuracy, respectively, compared to a model that does not take facial expressions into account. In ASD, this indicates that modeling is effective, which weights the output of each image.

## 1. Introduction

Experienced psychiatrists identify people with autism spectrum disorder (ASD) and schizophrenia (Sz) through interviews based on diagnostic criteria, their responses, and various neuropsychological tests (1). To improve the clinical diagnosis of neurodevelopmental disorders such as ASD and Sz, the discovery of disorder-specific biomarkers and behavioral indicators with sufficient sensitivity is important. Neurocognitive mechanisms of ASD and Sz are reviewed, where similar social cognitive deficits are observed, but neurocognitive processes are concluded to be different (2, 3). Among various indicators, eye movement, which can be easily obtained, has attracted much attention and various studies have been conducted for ASD and Sz (4, 5). ASD and Sz have abnormalities in cognitive functions, particularly social cognitive functions. Facial expression recognition is one of the social cognitive functions, and abnormalities have been reported in ASD and Sz (6, 7). People with ASD have been found to differ in the recognition of subtle emotional expressions and be less accurate when processing such basic emotional expressions as disgust, anger, and surprise (8). In addition, abnormalities in eye movement during facial expression recognition have also been reported (9). We hypothesized that if we could deepen our investigation of this trait in ASD, we would get more accurate classification. On the other hand, while there are papers that discuss differences in eye movements using other tasks between ASD and Sz, to our knowledge there are no papers that discuss differences in measured eye movements during facial expression recognition (5). Since ASD and Sz are often difficult to differentiate, we propose to examine eye movement differences during facial expression recognition for the distinction. ASD is a neurodevelopmental disorder, meaning that it does not appear in adulthood but is an innate trait (10). On the other hand, in clinical situations, some have spent their childhood without being diagnosed with ASD. But, in adulthood, difficulties in real social life may become emerged. In some cases, secondary disorders such as depressive state are shown, then the diagnosis of ASD is made when the first visit to a psychiatric hospital. Investigating the developmental trajectory in processing emotional stimuli in neurodevelopmental conditions is important. Based on the above, we investigate whether abnormalities in gaze activity of facial expression recognition are markers specific to ASD. In recent years, studies have been conducted using deep learning to make more accurate predictions. Cilia et al. have classified ASD by inputting eye movements during free viewing of various photos into a deep learning model (11). Li et al. used long short-term memory (LSTM) to classify the ASDs and controls by a model that considers the time series nature of eye movements (12).

In this paper, we describe previous studies that attempted to use eye movement to discriminate among psychiatric disorder groups. First, we present relevant studies on detection between ASD and control groups. Basic research has shown that facial scanning patterns are different between ASD and control groups (6, 9, 13). Further research has also been conducted into differences in facial expressions, reporting that the fixation time of infants’ eyes changes when viewing fearful faces (14). Król et al. used machine learning to reveal differences in gaze scanning patterns between ASD and control participants during facial stimuli (15). The scan-path length is also useful for classifying ASD and control individuals (16). Li et al. attempted to predict ASD by acquiring eye movement while showing 64 children a variety of tasks (17). Based on the premise that social stimuli are effective for discriminating ASD, Jiang et al. conducted a dynamic affect recognition evaluation task in which participants gradually increased their facial expressions from a state of no expression through stopping when their facial expressions were recognized (18). The results showed that ASD can be detected by random forest modeling using eye movement during a task. Another study used facial expression recognition tasks to classify ASD and control individuals (19). In recent years, research has further improved the accuracy by electroencephalogram in addition to eye movements (20, 21).

Next, we present studies that attempt to differentiate Sz from control individuals using eye movement. Morita et al. used eye movement during a smooth pursuit task to discriminate between Sz and control participants. The scan-path length is also an effective feature that separates two groups in free-viewing tasks (22, 23). The scan path consists of a series of fixations in which the gaze stops for a short period of time (typically 200-300 ms), during which there is a fast saccade motion. Loughland et al. showed differences in fixation patterns for faces in Sz and control groups using images of negative and positive facial expressions (7). Kacur et al. used eye movement during Rorschach tests (for mental discrimination) and analyzed them using various machine learning methods. Their results showed that the best classification results for Sz and control were obtained by constructing a convolutional neural network (CNN) model with a heat map showing eye-movement pauses (24).

As mentioned above, each disorder group has different gaze scanning patterns depending on the facial expressions shown in the facial expression recognition task (7, 14). Although basic research has shown that eye movements differ in disorder groups for each presenting emotion, machine learning models that take this into account have not been examined. Therefore, we propose a modeling method that takes into account the differences in gaze scanning patterns for each facial expression. We used machine learning to model the specificity of eye movements depending on the facial expression presented.

Our contribution is the comparison of facial expression recognition by disorder groups and modeling that takes into account the specific eye movement of each facial expression. We used the facial emotion identification test (FEIT) (25, 26). We provide a comprehensive analysis using eye movement during the FEIT for ASD and Sz in adults and children.

Section 2 describes the participants, the tasks used, and the experimental conditions, followed by statistical analysis. Section 3 describes the model used in this study. Section4 presents the results of the actual classification problems. Section 5 provides a comprehensive discussion of the results obtained, the limitations of this study, and future perspectives, followed in the final section by a comprehensive summary of the paper.

## 2. Data collection and basic analysis

### 2.1. Participants

We obtained eye movement data for adults with ASD and Sz and for children with ASD. No control participants had a history of psychiatric disorders, drug abuse, or epilepsy. No controls had lerning delays and communication problems because physicians and psychologists interviewed them and excluded. For each participant, the diagnosis was determined according to DSM-5 research criteria for schizophrenia or ASD. The ASDs were re-evaluated using the Autism Disorder Observation Schedule-2nd Ed. (27). The Szs were re-evaluated using the positive and negative syndrome scale (28) for schizotypy symptoms. the All data collection processes were approved by ethical committees at Nara Medical University and Nara Institute of Science and Technology. At the beginning of the recording, we explained the procedure to the participants and obtained informed consent.

#### 2.1.1. Dataset 1: Adults

For the adults, we collected data from 15 participants with ASD, 15 participants with Sz, and 26 participants as controls. For the whole group, we obtained Kikuchi’s Scale of Social Skills: 18 items (Kiss-18) (29) and the FEIT (25). For the ASD and control groups, we obtained the Social Responsiveness Scale Second Edition (SRS-2) (30). We did not obtain SRS-2 because we haven’t yet validated it for Sz. We have not taken SRS-2 because it has not been validated for Sz. For the ASD group, we obtained ADOS-2 (27). The Sz group was also assessed with PANSS (28) for schizotypy symptoms. We used the SRS-2 scores to determine the number of under-sampled eye movements (31) as a simple test of social functioning, as it is related to eye movement. We aligned the number of participants among all the groups using the SRS-2 scores of 16 participants (8 males and 8 females) in descending order, and the data from 15 ASD participants (9 males and 6 females) and 15 Sz participants (7 males and 8 females). Table 1 shows the participants’ details. These values are total raw scores.

**Table 1:** Details of adult participants.

Group	N (Males: Females)	Age	kiss-18	SRS-2	FEIT	ADOS-2	PANSS
Controls	16 (8:8)	29.3±3.72	64.7±11.0	55.2±20.9	15.5±2.07	N/A	N/A
ASD	15 (9:6)	32.1±9.13	45.3±9.46	73.3±36.0	14.5±2.97	46.5±11.9	N/A
Sz	15 (7:8)	26.0±5.85	59.1±9.14	N/A	14.3±2.52	N/A	67.1±13.0

#### 2.1.2. Dataset 2: Children

For the children, we collected data from 15 participants with ASD and 17 participants as controls. The SRS-2, FEIT, and attention-deficit hyperactivity disorder (ADHD) Rating Scale (ADHD-RS) (32) were obtained for the entire group and the Child Behavior Checklist (CBCL) (33) was obtained for the ASD group. We also aligned the number of participants between two groups using the SRS-2 scores of 15 participants (7 males and 8 females) in descending order and the data from 15 ASD participants (13 males and 2 females). Table 2 shows the participants’ details. These scores are total raw scores.

**Table 2:** Details of children participants.

Group	N (Males: Females)	Age	SRS-2	FEIT	ADHD-RS	CBCL
Controls	15(7:8)	10.0±1.55	19.2±11.5	22.1±2.85	6.88±9.40	N/A
ASD	15(13:2)	10.9±1.39	74.7±26.9	22.2±3.72	17.6±10.2	37.1±14.6

### 2.2. Procedure

**Figure 1:**
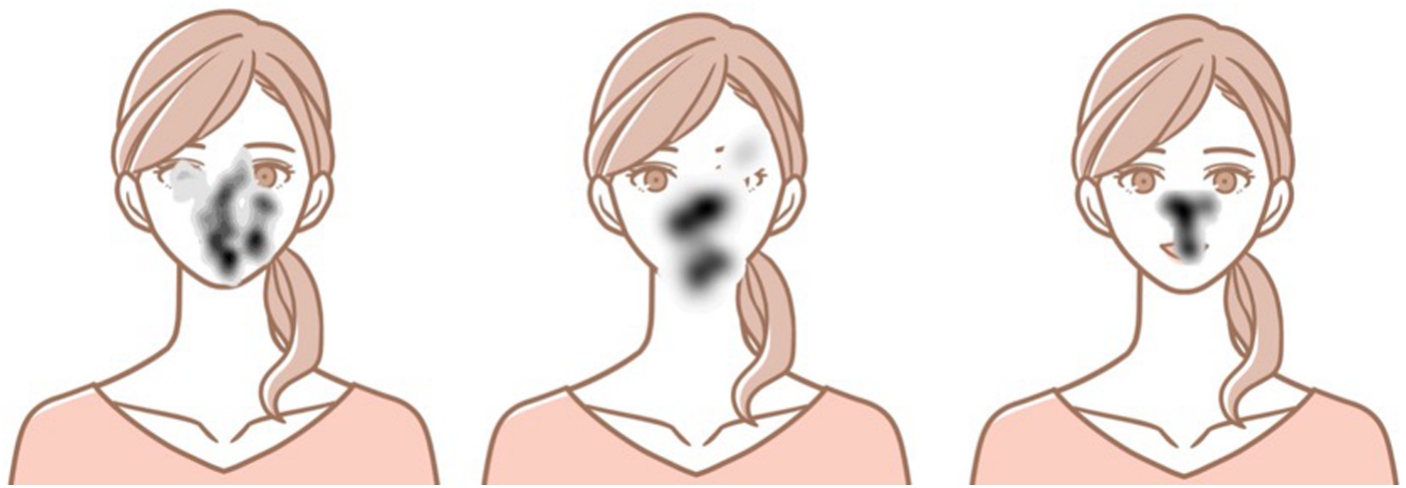
Schematic image and typical eye movement patterns. Left: ASD; center: Controls; right: Sz.

**Figure 2:**
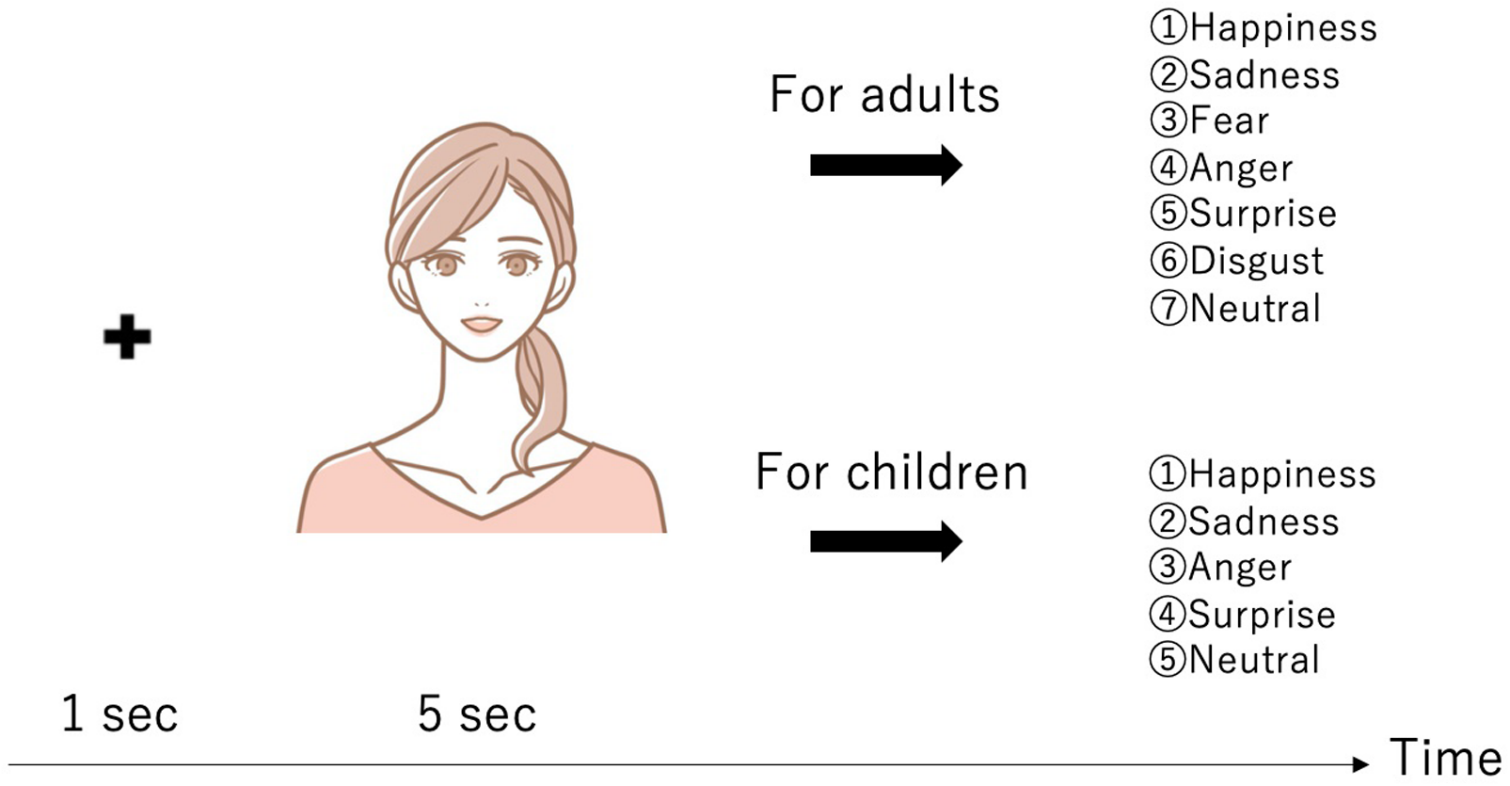
Experimental procedure: The adults were asked to choose from seven emotions (Happiness, Sadness, Fear, Anger, Surprise, Disgust, and Neutral), while the children were asked to choose from five emotions (Happiness, Sadness, Anger, Surprise, and Neutral). The actual images could not be depicted due to the FEIT’s copyright. Therefore, the images shown are schematic.

We used the FEIT (25, 26) as an emotion recognition stimulus to measure the facial expression recognition ability of the adults and children. The FEIT uses a morphing technique and includes facial expression recognition stimuli of various difficulty levels, such as strong or weak facial expressions. The procedure is shown in Figure 2. Figure 1 shows a schematic diagram of the tasks and typical eye-movement patterns for each group. ASDs have more fixation of the overall face, and Szs have less fixation at the eyes than the controls. First, the cross-shaped stimulus is shown for a second to concentrate on a display. The FEIT is then presented, displaying for 5 seconds, after which the participants are asked to verbally describe the facial expressions. For the adults, three images for each emotion (happiness, sadness, fear, anger, surprise, disgust, and neutral) were randomly displayed for a total of 21 images. For the children, a total of 32 images, 8 for each emotion (happiness, sadness, anger, and surprise) were randomly displayed. The FEIT scores were calculated as the number of correct answers (0 to 21 or 32) for each task.

We used Tobii Pro Fusion to measure the eye movements. The participants sat about 65 cm from the display, which had a sampling rate of 120 Hz and a resolution of 1920×1080.

### 2.3. Statistical analysis

We conducted an eye-tracking experiment to investigate the atypical gaze patterns of the ASD and Sz participants. The statistical analyses were carried out before the main study. We used fixation, saccade, and scan-path length for statistical analysis of the eye movements. Past studies have shown that these features differ in ASD and Sz individuals (16, 18, 23). We analyzed each facial expression presented in the FEIT and found significant differences in several features. We describe these features below.

#### 2.3.1. Fixation

Fixation is a movement that stops a gaze at a specific location. It is a slower, subtler movement to align the eyes with the target and prevent perceptual fading, with fixation duration ranging from 50 to 600 ms. The minimum duration required for information intake depends on the task and stimulus. This feature was calculated using attention filter in Tobii Pro Lab (Ver. 1.145) (34). We used the Velocity-Threshold Identification (I-VT) fixation classification algorithm, which is a velocity-based classification algorithm (35) that categorizes fixation and saccade based on velocity. If the velocity exceeds 100${}^{\circ }$/s, it is classified as saccade; otherwise, it is classified as fixation.

#### 2.3.2. Saccade

Saccade is fast eye movement in which both eyes move in the same direction and are induced spontaneously or involuntarily. The time to plan the saccade (latency) depends on the task and varies between 100–1000 ms with an average duration of 20–40 ms. This feature was calculated using Tobii Pro Lab (Ver. 1.145)’s attention filter (34).

#### 2.3.3. Scan-path length

The scan-path length is the average distance of an eye movement per sample. We calculated this feature using Python. Algorithm 1 is the pseudo code for calculation of the scan-path length. First, when either the right or left eye is detected, we compute and sum the distances between two consecutive samples. If neither eye is detected, we store the distance to it. The same calculation is repeated. Finally, the total is calculated and divided by the number of line segments to calculate the scan-path length.

**Algorithm 1:** Calculation of scan-path length

**Require:** Gaze samples each trial **Ensure:** Scan-path length scan-path length, line count, line length, length temporary ← 0 **for** A gaze sample to whole gaze sample **do** **if** A gaze sample is valid **then** Increased line count **if** Next gaze sample is invalid then Add line length to the length list and initialize **end if** length temporary ← Calculate a length of two samples scan-path length ← scan-path length + length temporary Initialized length temporary **end if** **if** A gaze sample is invalid **then** Add line length to the length list and initialize **end if** **end for** scan-path length ← Divide the sum of the line list by the line count

#### 2.3.4. Analysis method

**Figure 3:**
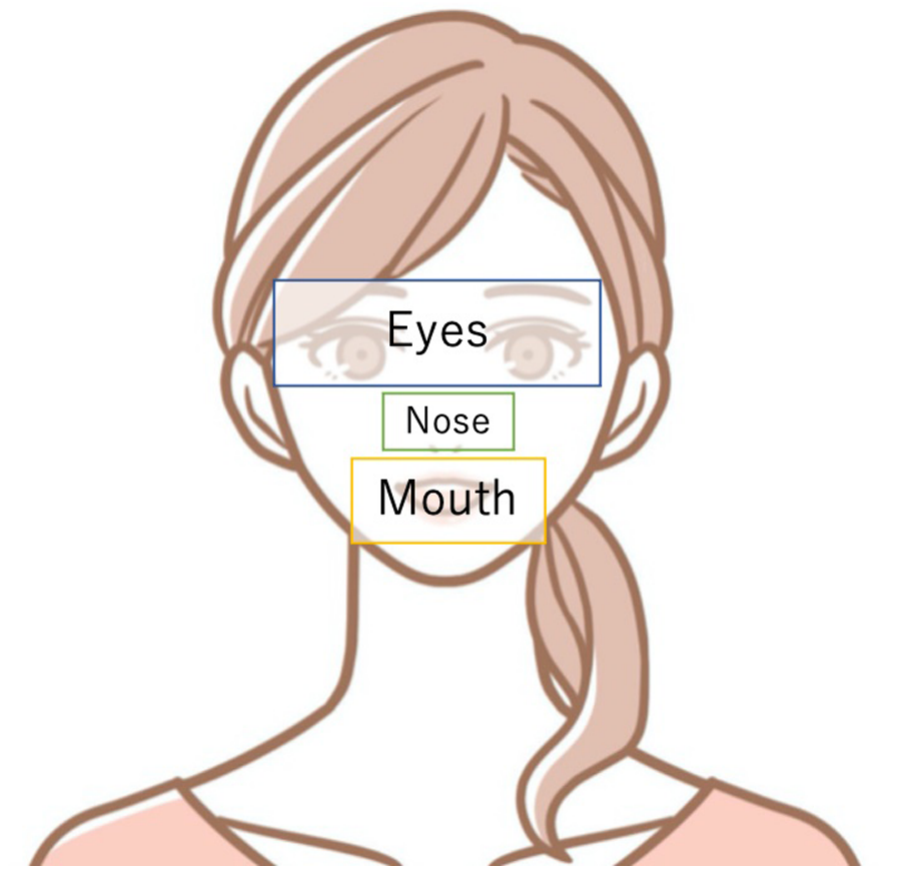
Schematic image of how AOI are set to the eyes, nose, and mouth. The eyes were set up to the eyebrows, with the nose and mouth surrounding them.

Some past studies set the Areas of Interest (AOI) at the forehead, both eyes, and the mouth (36); others set them at both eyes, the nose, the mouth, and the contour (37). Figure 3 shows how AOI is set to the eyes, nose, and mouth. Since the size of the mouth differs depending on the expression of the presented image, we changed the AOI for each image so that the entire region was included in each image. We excluded trials with a scan-path length of 0 because they do not correctly measure eye movements. In addition, all the data used in the analysis retained at least 40% of the gaze sample. We then analyzed 105 surprise trials, 101 happiness trials, 101 anger trials, 111 sadness trials, 101 neutral trials, 104 fear trials, and 107 disgust trials in the adults and 172 surprise trials, 154 happiness trials, 164 anger trials, and 164 sadness trials in the children. With the adults, we made multiple comparisons with Welch’s t-test for each feature with the control and ASD groups and with the control and Sz groups for each emotion in the FEIT. With the children, we used Welch’s t-test between the groups. We used the Benjamini and Hochberg method for correction (38).

#### 2.3.5. Statistical test result

**Figure 4:**
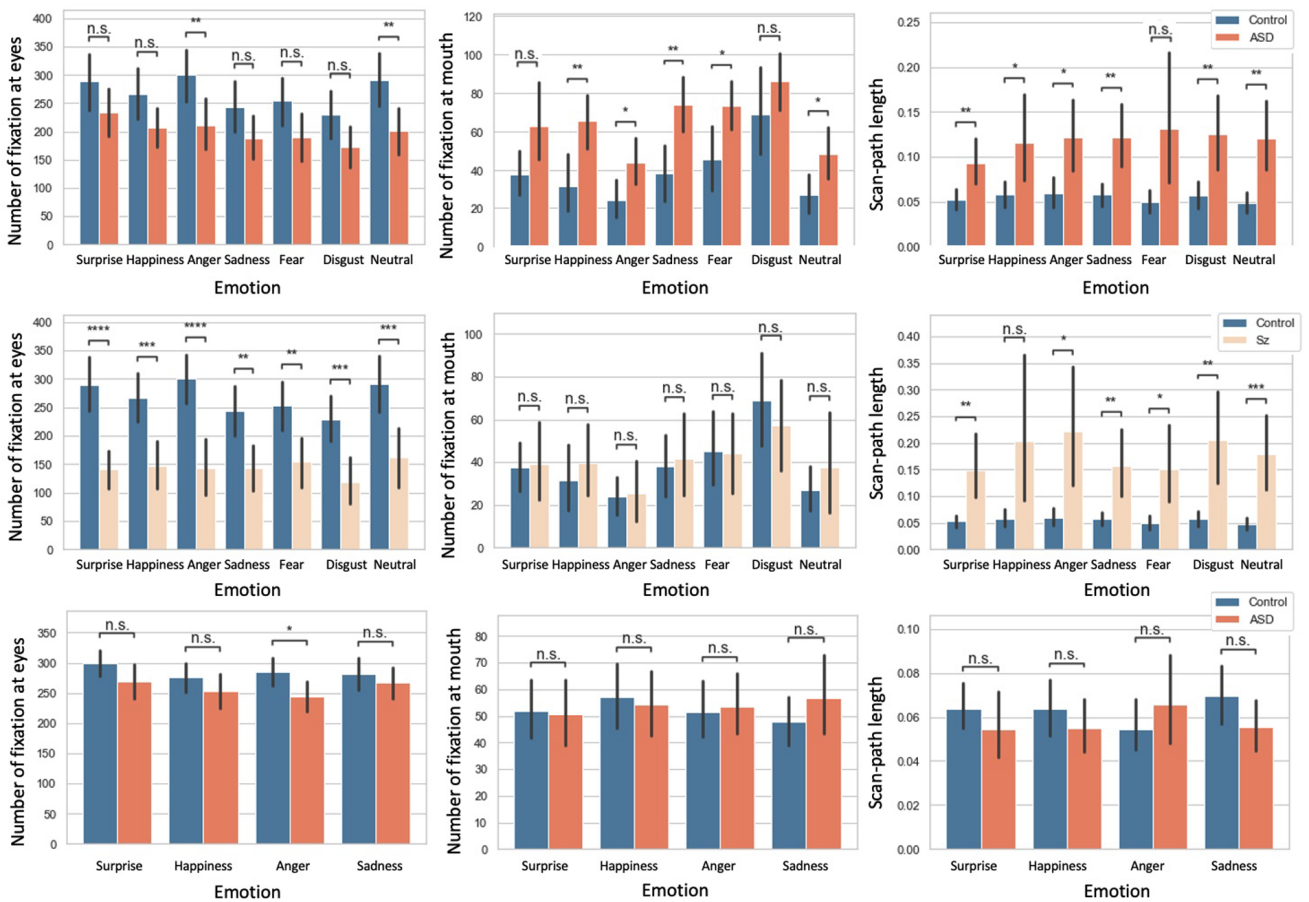
First row: comparison of ASD vs. control groups in adults; Second row: comparison of Sz vs. controls in adults. Third row: comparison of ASD vs. controls in children. The error bar denotes the size of the confidence intervals (95%), n.s.: no significance, *: p<0.05, **: p<0.01, ***: p<0.001, ****: p<0.0001.

Figure 4 shows a barplot for the number of fixations at the eyes and mouth, and the scan-path length (n.s.: no significance, *: p<0.05, **: p<0.01, ***: p<0.001, ****: p<0.0001). We also analyzed the number of fixations at the nose and the saccade. The results are contained in the supplemental materials.

We begin by describing the results for the adults. Table 3 shows the mean and standard deviation of each feature. For fixation at the eyes, between the control and ASD group, there were no significant differences for the surprise, happiness, sadness, fear, and disgust trials. However, for the anger and neutral trials, there were significant differences (p<0.01) between the control and Sz groups, and there were significant differences for all FEIT for each emotion (surprise and anger: p<0.0001; happiness, neutral, and disgust: p<0.001; sadness and fear: p<0.01). For the number of fixations at the mouth, there were no significant differences between the control and ASD groups for the surprise and disgust trials. However, for the happiness, anger, sadness, neutral, and fear trials, there were significant differences (happiness and sadness: p<0.01; anger, neutral, and fear: p<0.05) between the control and Sz groups. There were no significant differences for all FEIT. For the scan-path length, between the control and ASD groups, there were significant differences except for the fear trials (surprise, sadness, and neutral: p<0.01; happiness and anger: p<0.05).

**Table 3:** Statistical analysis of control, ASD, and Sz adult participants (mean±SD).

	Controls	ASD	Sz
	Surprise (105 trials)
Number of fixations at eyes	288.33±149.18	234.23±125.23	140.35±107.55
Number of fixations at mouth	37.64±36.27	62.63±60.94	39.00±56.28
Scan-path length	0.05±0.04	0.09±0.08	0.15±0.19
	Happiness (101 trials)
Number of fixations at eyes	266.68±131.35	206.18±108.08	146.67±128.70
Number of fixations at mouth	31.62±44.68	65.21±41.92	39.79±49.58
Scan-path length	0.06±0.05	0.12±0.15	0.20±0.42
	Anger (101 trials)
Number of fixations at eyes	299.69±139.58	210.11±131.65	143.73±140.49
Number of fixations at mouth	23.94±29.68	43.80±36.87	25.50±41.67
Scan-path length	0.06±0.05	0.12±0.13	0.22±0.33
	Sadness (111 trials)
Number of fixations at eyes	243.73±150.27	187.35±123.43	142.50±115.62
Number of fixations at mouth	37.95±48.48	73.67±46.99	41.63±54.69
Scan-path length	0.06±0.04	0.12±0.12	0.16±0.19
	Neutral (101 trials)
Number of fixations at eyes	291.38±151.09	200.11±129.89	161.62±140.37
Number of fixations at mouth	27.05±31.71	48.09±40.15	37.48±65.84
Scan-path length	0.05±0.04	0.12±0.12	0.18±0.19
	Fear (104 trials)
Number of fixations at eyes	253.91±129.23	189.00±127.43	153.38±131.99
Number of fixations at mouth	45.12±50.76	73.17±40.50	43.88±53.44
Scan-path length	0.05±0.04	0.13±0.24	0.15±0.22
	Disgust (107 trials)
Number of fixations at eyes	228.74±125.74	172.00±121.02	118.41±120.88
Number of fixations at mouth	68.74±66.18	86.21±46.43	57.21±66.96
Scan-path length	0.06±0.04	0.13±0.14	0.20±0.28

Turning our attention to the children’s results, Table 4 shows the mean and standard deviation of each feature. For the number of fixations at the eyes, there was a significant difference for the anger trials (p<0.05), but there were no significant differences for the others. For the number of fixations at the mouth and the scan-path length, there were no significant differences for all trials.

**Table 4:** Statistical analysis of controls and ASD children participants (mean±SD).

	Controls	ASD
	Surprise (172 trials)
Number of fixations at eyes	300.01±117.31	268.62±119.46
Number of fixations at mouth	51.76±54.53	50.81±51.47
Scan-path length	0.06±0.05	0.05±0.07
	Happiness (154 trials)
Number of fixations at eyes	275.83±114.19	253.12±124.58
Number of fixations at mouth	57.28±55.23	54.22±53.33
Scan-path length	0.06±0.06	0.05±0.05
	Anger (164 trials)
Number of fixations at eyes	285.29±116.30	243.84±106.33
Number of fixations at mouth	51.50±51.39	53.64±49.30
Scan-path length	0.05±0.05	0.07±0.09
	Sadness (164 trials)
Number of fixations at eyes	280.54±125.82	267.56±112.78
Number of fixations at mouth	47.78±46.57	56.52±65.67
Scan-path length	0.07±0.06	0.06±0.05

## 3. Modeling and evaluation

**Figure 5:**
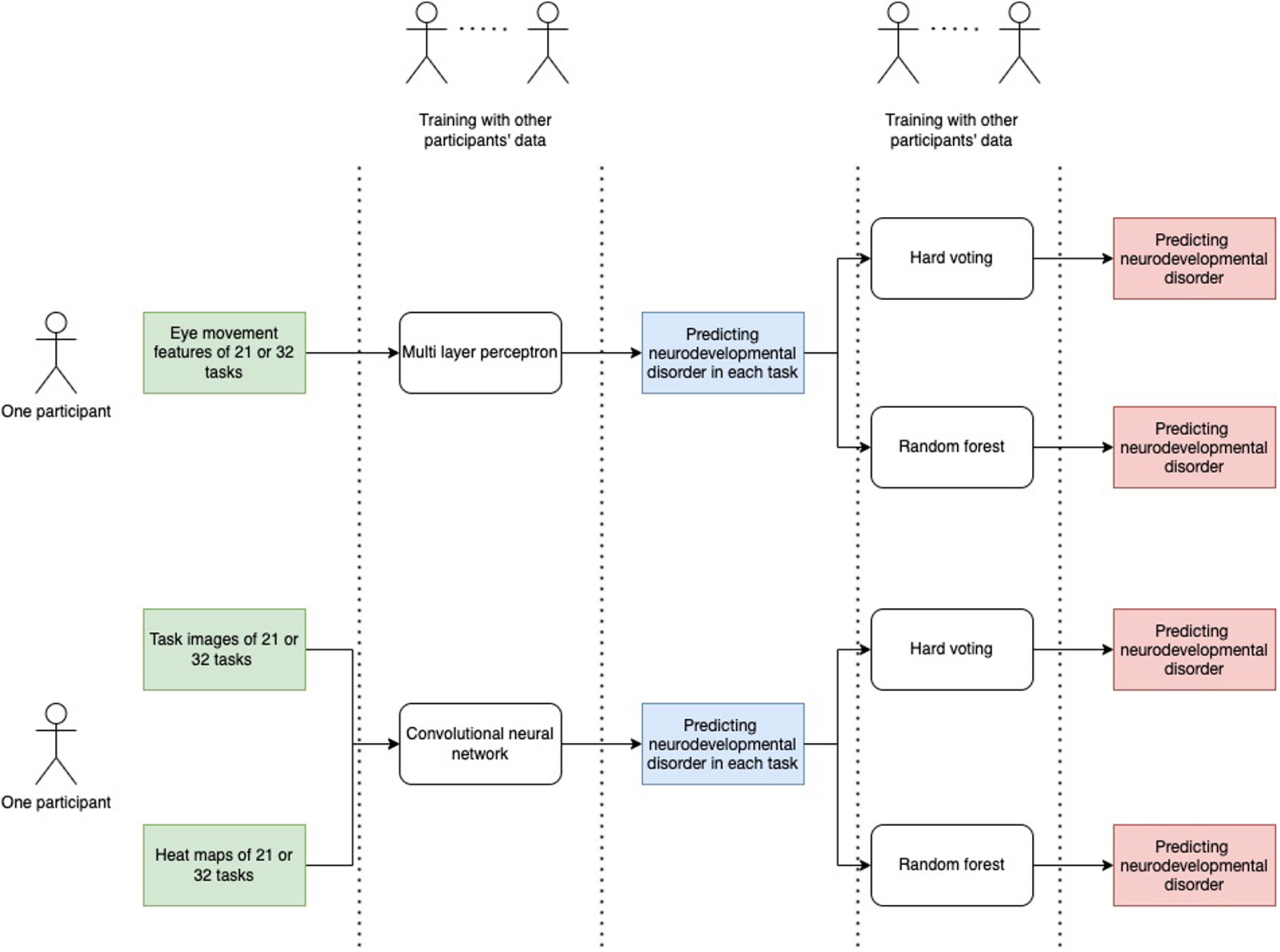
Flow of classification models.

We propose a model that classifies disorder groups by weighting each task, as shown in Figure 5. We used a baseline model and a CNN model. In each case, we used hard voting and random forest for a total of four models for comparison. Hard voting denotes the predicted class labels for majority rule voting. We will now explain the details of each model.

### 3.1. Baseline model

The features used for the baseline multi-layer perceptron are the number of fixations, the number of saccades, and the scan-path length. The baseline model uses a structure with three hidden layers, the maximum number of epochs for both models is 300, and early stopping is applied if the validation loss is not updated for 15 epochs. We used Adam (39) as the optimization method and cross-entropy loss as the loss function. The learning rate was optimized by nested leave-one-participant-out cross-validation. The number of splits was set to 1 for the test data and 8:2 for the training and validation data. The sample size for each learning was 630 or 651, which is sufficient compared to the previous study (24). We used PyTorch (40) for implementation. The details of the structure are shown in Table 5. 21 or 32 tasks were binary classified and input to hard voting or random forest, which is our contribution to consider the importance of tasks by random forest to determine the final decision for considering the influence of the FEIT emotion.

**Table 5:** Details of model structure.

	Baseline	CNN
Feature	Number of fixations	Heat map
	Number of saccades	Task image
	Scan-path length	
Structures	[5,5], [5,5], [5,5]	[3×3, 3×3; 8,16]
Baseline: [neurons]		
CNN: [kernel sizes]	kernels]	

### 3.2. CNN model

The CNN features are the task images and the heat map of eye pauses, which can be input simultaneously to explicitly learn the facial regions and expressions. For the heat map, the pixels recorded in the eye-movement data were set to 1 and the other pixels were set to 0. Then, the image was blurred using a Gaussian kernel (σ=10). A schematic diagram of the heat map superimposed on the task images is shown in Figure 1.

Because our data set size was small, we trained it in the following three steps:

∙ After cropping the center, the image size was downscaled to a 34*34 grayscale.
∙ The number of layers in the CNN was reduced (2 layers + 1 fully connected layer).
∙ We performed leave-one-participant-out cross validation to ensure the training data size.

**Figure 6:**
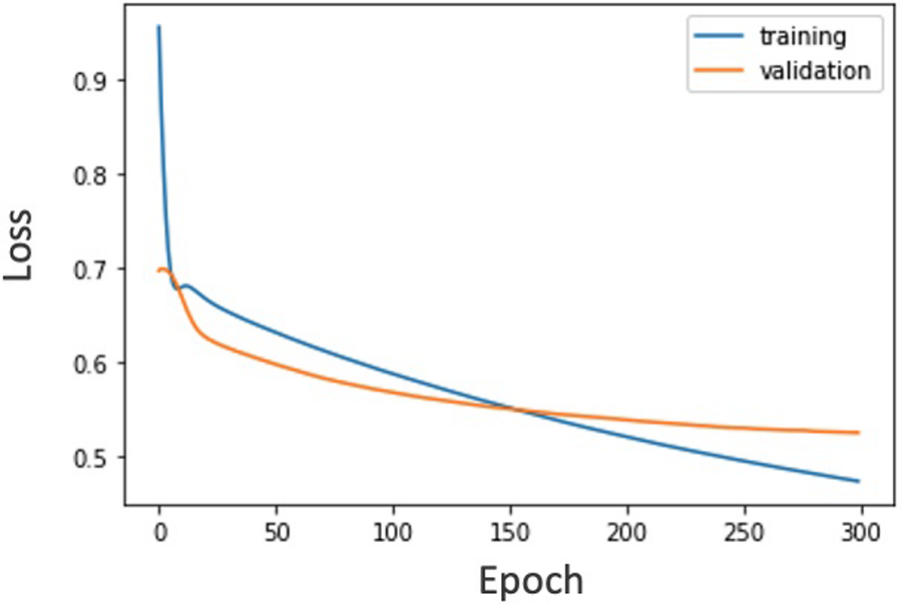
Loss during training of the CNN in the Sz and control group classification.

The maximum number of epochs for both models is 300 and early stopping is applied if the validation loss is not updated for 15 epochs. We used Adam (39) as the optimization method and cross-entropy loss was used as the loss function. The learning rate was optimized by nested leave-one-participant-out cross-validation. The number of splits was set to 1 for the test data and 8:2 for the training and validation data. Since the sample size for each learning was 630 or 651, which is sufficient compared to a previous study (24), we did not consider it necessary to use such traditional approaches as a support vector machine. For reference, the loss during training of the CNN in the Sz and control group classifications is shown in Figure 6. This is a learning curve, and if the loss is falling, it means that learning is progressing. In other words, the model can classify Sz and control for the training and validation data. Although it has a small data size, it shows that the learning is progressing. We used PyTorch (40) for the implementation. The details of the structure are shown in Table 5. 21 or 32 tasks were binary classified and input to hard voting or random forest, which is our contribution to consider the importance of tasks by random forest and determine the final decision for considering the influence of the FEIT emotion.

### 3.3. Evaluation

We used nested leave-one-participant-out cross-validation to evaluate our experiments. In each run, one participant was left out as testing data, while the rest were used for training. The testing results of all the participants were combined and evaluated for accuracy, sensitivity, and specificity. The evaluation indices were true positive (TP), true negative (TN), false positive (FP), and false negative (FN). The disorder groups were designated as positive and the controls as negative:

(1) $$Accuracy=\frac{\mathrm{TP}+\mathrm{TN}}{\mathrm{TP}+\mathrm{FP}+\mathrm{TN}+\mathrm{FN}}$$

(2) $$Sensitivity=\frac{\mathrm{TP}}{\mathrm{TP}+\mathrm{FN}}.$$

(3) $$Specificity=\frac{\mathrm{TN}}{\mathrm{TN}+\mathrm{FP}}$$

## 4. Results

The results are shown in Table 6. The classification results of the adult ASD and control participants are shown first. Among the four models, our proposed model, the weighted CNN, had the best accuracy and there was a significant difference in the binomial test by chance rate (p<0.05). The weighted model increased the specificity from 0.625 to 0.750, which improved the baseline accuracy. Next, we show the classification results for the Sz and control groups. Compared with the four models, the CNN and weighted CNN have the highest accuracy. In this case, there was no improvement due to random forest. A binomial test with chance rate showed a marginally significant difference (p=0.052). Finally, the classification results of the ASD and control groups of the children show that, among the four models, our proposed weighted-CNN model and the weighted baseline had the best accuracy, and there was a significant difference in the binomial test by chance rate (p<0.05). Random forest increased the sensitivity from 0.333 to 0.667, which improved the baseline accuracy, and also from 0.286 to 0.600, which improved the CNN accuracy.

**Table 6:** Results of classification of ASD and controls in adults, Sz and controls in adults, and ASD and controls in children: Best results are shown in bold.

	Accuracy	Sensitivity	Specificity
**ASD and controls in adults**			
Baseline	0.419	0.267	0.563
Weighted baseline	0.645	0.600	0.688
CNN	0.613	**0.800**	0.438
Weighted CNN	**0.710**	0.733	**0.688**
**Sz and controls in adults**			
Baseline	0.613	0.467	**0.750**
Weighted baseline	0.548	0.467	0.625
CNN	0.645	**0.733**	0.563
Weighted CNN	**0.645**	0.667	0.625
**ASD and controls in children**			
Baseline	0.467	0.333	0.600
Weighted baseline	0.667	**0.667**	0.667
CNN	0.500	0.286	0.667
Weighted CNN	**0.667**	0.600	**0.733**

## 5. Discussion

### 5.1. Principal findings

In this study we obtained eye movements during the FEIT in children, adults with ASD, and adults with Sz, and analyzed the relationship between eye movement and neurodevelopmental disorder groups using statistical analysis with reference to previous studies. We solved the classification problem for the control groups and each disorder group by machine learning using eye movement. In doing so, we performed modeling utilizing the knowledge that the eye movements of the disorder group differ from those of the controls depending on the facial expressions that the disorder group sees, which had been analyzed in the basic research, and compared the results with those of a model that does not consider differences in facial expressions. Our proposed weighted-CNN model had the best accuracy for the problem of classifying adults with ASD and the control group, 71.0%, and was 10% more accurate than the model that did not account for facial expressions. Compared to the chance rate, this was a statistically significant difference by the binomial test (p<0.05). It also showed the best results for the problem of classifying the Sz and control participants in the adults and the ASD and control participants in the children. Accuracy was 64.5% (p=0.052) and 66.7% (p<0.05), respectively. There was no difference in accuracy for Sz compared to the model without facial expressions, but there was a 16.7% improvement for ASD in the children. These results are discussed in conjunction with the statistical analysis results.

### 5.2. Effects of specificity of eye movements with presented facial expressions for each group

**Figure 7:**
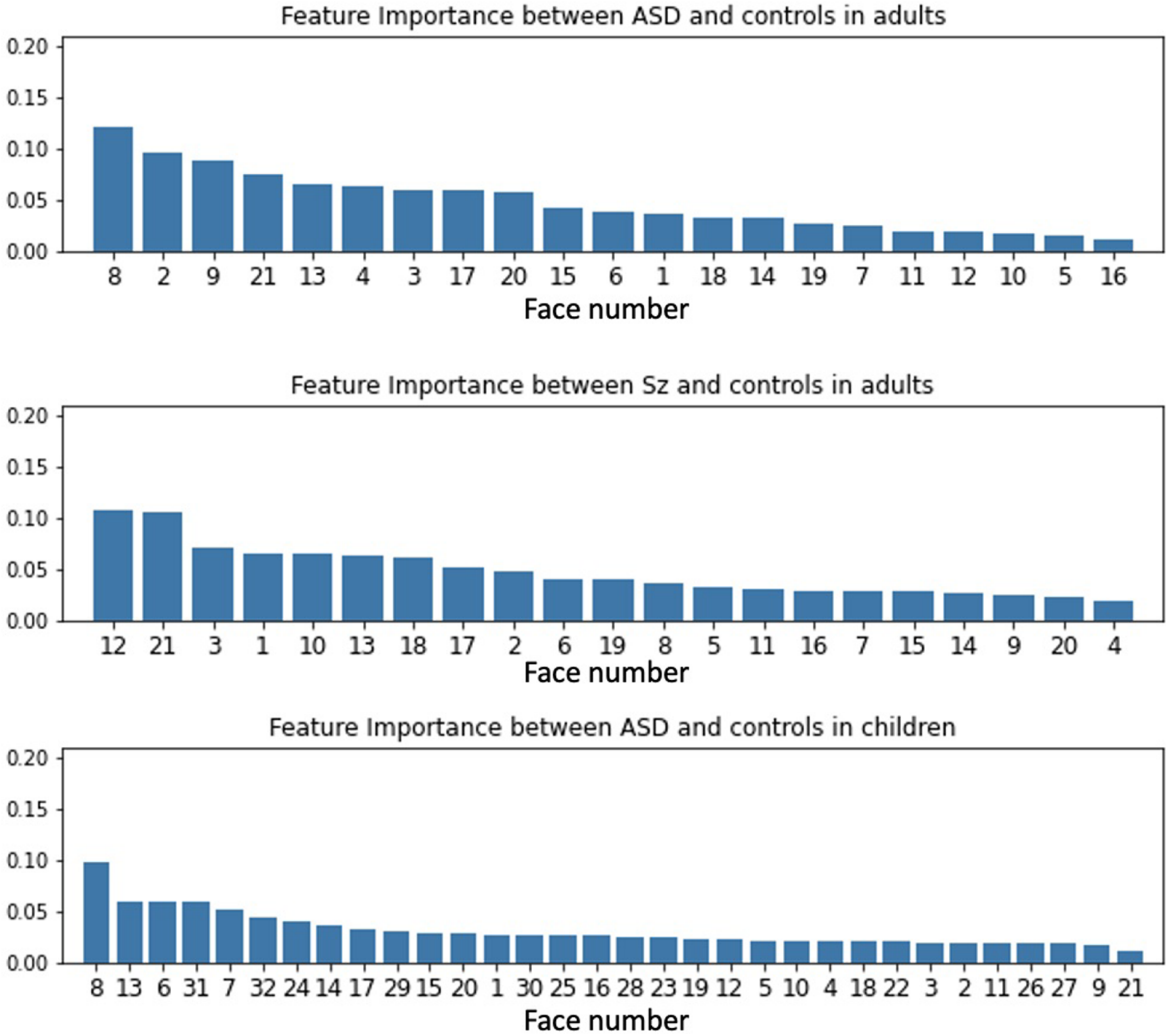
Feature importance for each weighted-CNN model.

The random forest classifier calculates the importance of features and determines how to partition the data into subsets to most effectively distinguish classes. We calculated feature importance for the most accurate CNNs weighted with random forests for the adult ASD and control models, adult Sz and control models, and child ASD and control models. We calculated the average of the importance of all cross-validations. In Figure 7, we show the importance of features. Table 7 shows the correspondence between each face number and the presented emotion.

**Table 7:** Correspondence between each face number and the presented emotion in FEIT.

Emotion	Face number
**FEIT for adults**	
Surprise	1, 7, 14
Happiness	3, 9, 11
Anger	13, 15, 21
Sadness	4, 16, 19
Neutral	2, 8, 17
Fear	5, 10, 20
Disgust	6, 12, 18
**FEIT for children**	
Surprise	3, 8, 10, 12, 19, 22, 25, 32
Happiness	5, 7, 9, 14, 21, 23, 26, 30
Anger	1, 6, 13, 15, 18, 24, 27, 29
Sadness	2, 4, 11, 16, 17, 20, 28, 31

#### 5.2.1. ASD and control adults

Between the ASD and controls in the adults, face 8 (neutral) had the highest score with face 2 (neutral) the next highest. The results of the statistical tests showed that there were significant differences in all three features for the neutral trials. Some individuals with ASD may have difficulty distinguishing subtle facial expressions (18). In addition, as reported in Figure 4, fixation to the eyes of a neutral face is different between adults with ASD and control. In the present results, the neutral face was given the highest weight in the random forest classification, which suggests that neutral face recognition may have been more specific to the classification of ASD and control in this study.

#### 5.2.2. Sz and control adults

For the Sz and controls, the accuracy was the same for each image with and without weights. The statistical analysis showed that there were statistically significant differences in fixation at the eyes regardless of which emotion was presented. Based on the above, the top two features of importance are face 12 (disgust) and face 21 (anger), but we do not think that feature importance has any particular significance. These results suggest that there is no difference in Sz by facial expression, but the face gaze scanning pattern may be different from that of the control group.

#### 5.2.3. ASD and control children

Finally, we discuss the results for ASD in children and the control group. Feature importance was highest for face 8 (surprise), followed by face 13 (anger) and face 6 (anger). The statistical tests showed that there was a significant difference only in fixation at the eyes compared to the anger trials. The 16.7% improvement in accuracy by weighting for each facial expression indicates that this modeling is also effective for ASD in children.

Compared to the adult results, the weighted models increased the accuracy of the adults and the children. Since the childhood version of FEIT used in this study does not include a neutral face, no direct comparison is possible. But it does include anger as a lower weight. Similarly, in the case of children, the emotion with the next highest weight after surprise is anger. In the statistical tests, Figure 4 shows that eye movements for control and anger are different for both groups of children, which suggests that a specificity might exist for eye movements for anger in both adults and children.

### 5.3. Error analysis

There were cases in which the proposed method did not work. There were 2 adults with ASD, 1 adult with Sz, an adult in the control group, and 4 children with ASD and one control child who could not be detected correctly by any of the 4 models constructed in this study. We checked the data for these individuals and noted that their gaze did not move from the center at all. Considering the possibility that they were not solving the task seriously, we checked the FEIT scores and found that their scores were not lower. The respective FEIT scores were 18 and 19 for the two adults with ASD, 15 for the adult with Sz, and 15 for the control. The 4 children with ASD scored 19, 25, 22, and 17 and the control had 23. This suggests that people with wide peripheral vision may be able to successfully complete the task without moving their eyes.

### 5.4. Limitation

A limitation of this study is the small data size. We need to increase the number of participants and conduct verification to ensure validity. In addition, error analysis showed that the method used in this study did not successfully detect people who observed objects through peripheral vision. Therefore, we will conduct experiments with a larger number of data in the future to investigate a method that can be used for people whose gaze does not move much.

As noted in the error analysis, we misidentified 2 ASD and 1 Sz in the adults and 4 ASD and 1 control in the children. As a percentage, we misidentified 6.5% of the subjects in adults and 17% in children. The inability to identify participants who solved the task without showing much eye movement is a limitation of the present models.

Last, in this paper we recruited all participants without intellectual difficulties. However, because we did not obtain any actual IQ values, this study does not adequately take into account the influence of intellectual level. This is a limitation because ASD and Sz includes a wide range (a spectrum) of symptoms, skills, and levels of disability. The participants of this study were only a small number of mild (high-functioning) cases, and it remains unclear whether all types of neurodevelopmental disorders have the same effect. We need to consider the relationship between the proposed model and the individual nature of disorders by obtaining actual IQ values and other relevant factors.

## 6. Conclusion

In this study, we examined whether the accuracy of classification could be improved by taking into account differences in eye movements due to facial expressions in the control, ASD, and Sz groups. The results showed that taking into account the differences in each image improved the accuracy of distinguishing between the control and ASD adults by 10%. The study also confirmed a 16.7% improvement in accuracy for children with ASD. Both results were significantly different compared to the chance rate (p<0.05). For the control and Sz participants, there was a marginally significant difference from the chance rate in the best model, but no improvement in accuracy. There was a difference in eye movement for each facial expression between the ASD and control groups, especially for weaker expressions. Sz and control participants showed differences in eye movement for each expression, but not for each presented emotion. In ASD, this indicates that modeling is effective, which weights the output of each image.

## Data Availability

The raw data supporting the conclusions of this article will be made available by the authors, without undue reservation.
